# Investigation of void content in *Borassus flabellifer* fiber/epoxy bio-nanocomposite using hyperparameter tuned ANN and response surface methodology optimisation

**DOI:** 10.1038/s41598-025-06740-0

**Published:** 2025-07-02

**Authors:** Suresh Thirupathi, Venkatachalam Gopalan, Elango Mallichetty

**Affiliations:** 1 School of Mechanical Engineering, Vellore Institute of Technology, Chennai, Tamil Nadu 600127 India; 2 Centre for Advanced Materials and Innovative Technologies, Vellore Institute of Technology, Chennai, Tamil Nadu 600127 India

**Keywords:** *Borassus flabellifer*, Nanofiller, Void content, Hyperparameter, ANN, DOE, Engineering, Materials science

## Abstract

Void development is one of the main problems faced by natural fiber polymer composites since it severely affects their physical and mechanical properties. It limits these composites for use in construction, aerospace, automotive and marine uses. Hence, this study takes on this issue by incorporating various nanosized Multi-Walled Carbon Nanotubes (MWCNT), hexagonal Boron Nitride (h-BN) and Alumina (Al_2_O_3_) nanofillers in epoxy-based *Borassus flabellifer* fiber (BFF) composites fabricated using the hand layup technique. The results show that increasing volume fraction of nanofiller gives way to decreasing fiber volume fraction, together with increase in composite density and void content. MWCNT-filled composites have the highest void content percentage among the different nanofillers investigated because of their lower theoretical density, which is inversely proportional to void content percentage. This research investigates the effects of the type of nanofiller, BFF mesh size and weight percent of the fiber upon the void content in fiber-reinforced composites. Design of Experiments approach is utilized to analyse the effect of these parameters and ANN model, employing advanced hyperparameter optimization strategy developed in Python, is used to elaborate upon specifics of the characteristics of void formation. Quantitative analysis of void content and particle distribution, analysed through SEM imaging and microstructural characterization through optical microscopy, further confirms these results, providing detailed information about void formation and filler dispersion. The optimized combination (1 wt% fiber content, 75 µm fiber mesh size and 1 wt% h-BN nanofiller) yielded 1.90% lowest void content after experimentation. This research provides fundamental understanding of the void mechanisms concerning bio-nano composites and presents an optimal predictor model that minimizes voids. This contributes and builds toward the directions of advancing materials for high-performance applications.

## Introduction

The natural fibers used in composite materials have increased attention due to their lightweight, eco-friendly and biodegradable characteristics, making them a more attractive alternative to synthetic fibers. Composite materials, especially polymer based biocomposites, exhibit remarkable mechanical and physical properties and are most suitable for a wide field of uses in the automotive, aeronautic and construction industries^1^. However, the presence of voids, microscopic air pockets within the composite, can significantly compromise these properties by reducing strength, stiffness and durability, while increasing susceptibility to moisture and crack propagation. Natural fibres have limited structural applications due to their limited compatibility with polymer matrices and significant water absorption. Surface treatment and nanofiller insertion significantly improve interfacial bonding, reduce moisture uptake and improve composite performance^2^. Several possible defects occur in the manufacture of polymer composites, including resin-rich zones, distorted/crimped fibers, foreign additions and spaces. Voids remain the extreme challenge because they occur most often in the corners of composite components. Vantage points are the most disconcerting because not only are they quite difficult to remove, but their influence is also very adverse on the mechanical performance of the substance^3^. Several factors contribute to the formation of voids in polymer composites. These include bubbles generated by the evolution of volatile by-products during the curing of the polymer matrix, the use of high-viscosity resins which cannot fully wet crowded fibers and incidental entrapping of air in the material system. Other contributory causes vary from fabrication defects, such as faulty vacuum bags to insufficient vacuum sources^4^. The Natural fiber-reinforced composites, despite their performance drawbacks owing to weak fiber-matrix bonding, have remarkable potential in industrial applications. Advanced methods involving surface treatments of fibers, hybridization and nanofiller incorporation are employed to enhance the material characteristics and strengthen the bonding^5,6^. Natural fiber surface characteristics, such as morphology, topography and chemical structure, are greatly modified by chemical treatments including acetylation, alkali, sodium chlorite, silane and benzoylation. NaOH treatment or mercerization is a simple yet highly effective technique for the removal of lignin, pectin, oil and wax. In addition to giving the fibers a good clean, this process provides a uniform, smooth and defect-free surface that offers improved performance in composite applications^7–9^. Nanofillers, enhancing fiber-matrix bonding in NFCs, cause a significant improvement in mechanical, thermal and tribological properties, given that they are evenly dispersed. Nanofillers, inorganic (Al_2_O_3_, h-BN, MgO, CNT, SiO_2_, ZnO, TiO_2_ and CaCO_3_) and organic (cellulosic fibers, graphite and graphene), provide a multitude of advantages. It is important to consider the proper choice of filler type and concentration such that they maximize performance while not interfering with the integrity of composites based on the types of fibers and matrices utilized. However, a lot of challenges lie ahead with the optimization of properties, particularly providing tough competition for filler contents, density and void generation within the same matrix^10–13^.

The application of Machine Learning (ML), particularly Artificial Neural Networks (ANNs), has gained traction in composite materials for enhancing modeling accuracy and optimizing design processes^14,15^. ANNs excel in predicting properties like void content and mechanical behavior, as shown in studies on jute fiber-reinforced concrete^16^, polytetrafluoroethylene composites^17^ and epoxy-based hybrid composites^18^. The application of ANN in polymer composites has been extended to tribological behavior analysis^19,20^, water absorption prediction^21^ and optimization of machining characteristics^22,23^.In the context of natural fiber polymer composites (NFPCs), ANN models have been utilized to assess mechanical performance^24^, shear properties^25^ and tribological behavior^26^, supporting our focus on bio-nanocomposites. For nanofiller-enhanced composites, ANN predicts physico-mechanical and thermal properties with Al_2_O_3_ and nanoclay^27,28^, inspiring our nanofiller parameter selection. Studies integrating ML with hyperparameter tuning^29^ refined our approach, optimizing ANN hyperparameters to enhance void prediction precision. Combined with response surface methodology (RSM) from prior optimization studies^30^, this methodology underpins our hybrid strategy to minimize void content in BFF/epoxy composites.

A study presented a Convolutional Neural Networks (CNN) based method for automatically assessing void content in composite laminates that outperformed existing image thresholding techniques and did not require calibration^31^. Artificial Neural Networks methodology based on machine learning has been used for calculating the transverse mechanical characteristics of unidirectional CFRP composites with micro voids, to reach the optimum void content for better performance and dependability^32^. Integrating ANN with numerical models made it possible to examine accurately for various conditions of strengthening substance. This approach effectively assisted in assessing flow front velocity and impregnation behaviour for better void content control. The extensive experimental data contributed to develop more efficient composite production technologies^33^. This study was to assess the influence of porosity on ultimate strength with the use of an ANN via backpropagation algorithm. The data for fatigue tests were split into training and validation sets, where porosity levels were incorporated as input. The LM learning algorithm showed satisfactory predictive capability for the ultimate strength, which thus served as an excellent guide for evaluating the performance of composites^34^. This study utilized RSM and ANN to optimize Porous Asphalt Mixture (PAM) design by predicting air voids and permeability with minimal trial batches. A total of 260 samples with varying aggregate proportions and compaction levels were analysed, achieving optimal permeability (> 0.12 cm/s) while ensuring mix stability. Results suggested coarse aggregate content of 60–90% and fines up to 20%, depending on compaction levels. The proposed approach improved efficiency, reduces material waste and enhances semi-flexible pavement design^30^.

This study investigates the impact of fibre weight percentage, fibre mesh size and nanofiller type (MWCNT, h-BN and Al_2_O_3_) on the void content of BFF-reinforced epoxy composites. Understanding void formation is critical for improving composite quality. To further the study, ANN prediction data is combined with response surface methodology (RSM) to determine the impact of these parameters on void content, offering significant insights for defect-free composite production.

## Materials and methods

### Material selection

*Borassus flabellifer* (Palmyra Palm) fruit fibers, sourced from the rich landscapes of Tirunelveli, Tamil Nadu, undergo meticulous preparation to serve as eco-friendly reinforcements in composite materials. The fibers are cleaned, air-dried and precision-cut into 20-mm length before being crushed and sieved into particles ranging from 75 to 225 microns. These finely prepared fibers and particles, bring natural strength to the composite by modifying the surface of the fiber using a 5% NaOH concentration^35^. The epoxy matrix binds the fibers and shields them from environmental exposure while evenly distributing loads to enhance overall performance. For this study, epoxy resin (LY556) and hardener (HY951), chosen for their superior compatibility and cost-effectiveness, are sourced from Herenba Instrument & Engineers in Chennai. Advanced nanofillers are incorporated to elevate the material’s properties. Multi-walled carbon nanotubes (MWCNT) and alumina are procured from Sisco Research Laboratories Pvt. Ltd, Mumbai and nano h-BN, known for its exceptional thermal and mechanical attributes, is supplied by Nanoshel, India. Together, these components promise a robust and innovative composite solution for modern engineering challenges.

### Bio-nanocomposite material preparation

**Fig. 1:**
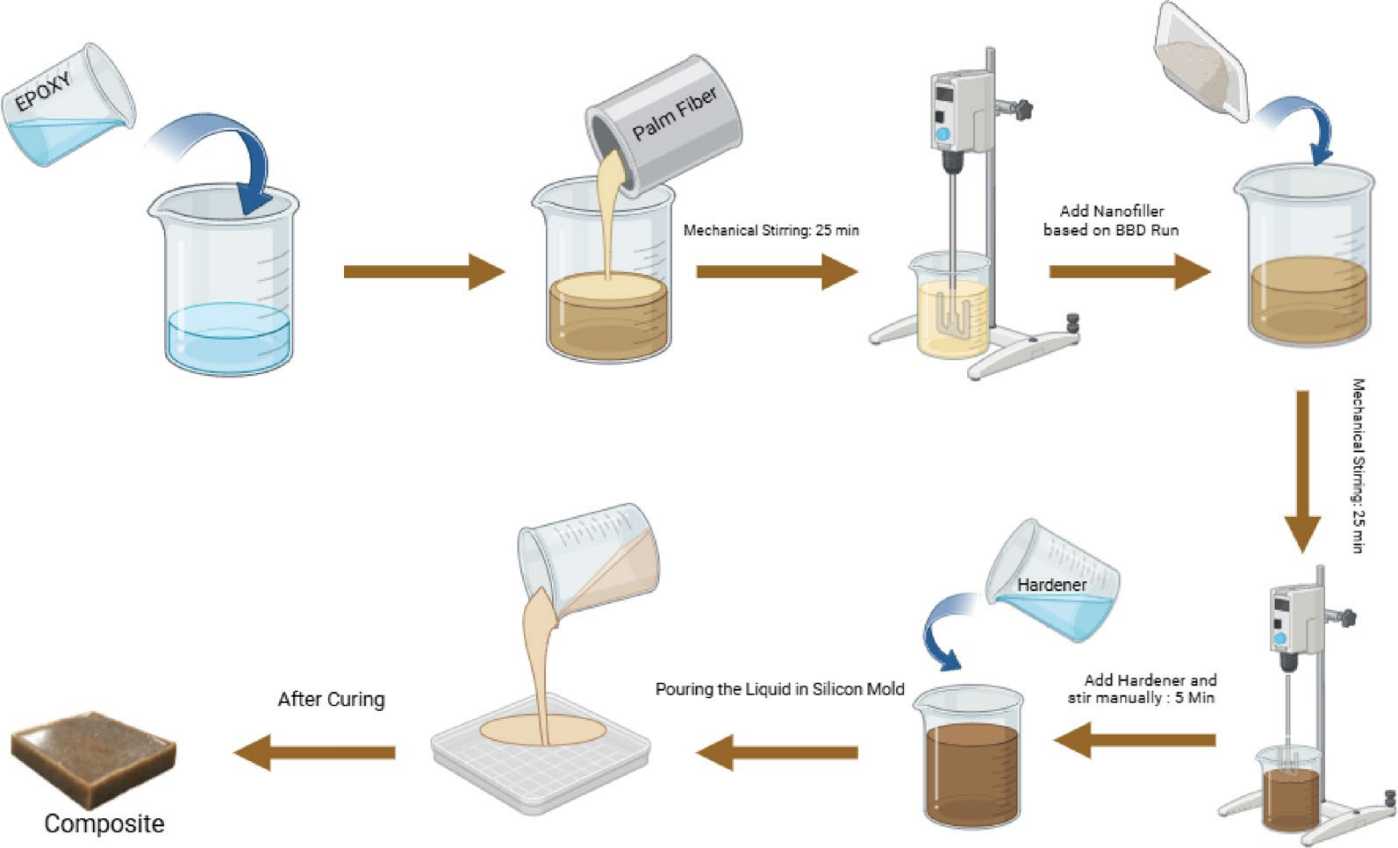
Fabrication method of composite material.

**Fig. 2:**
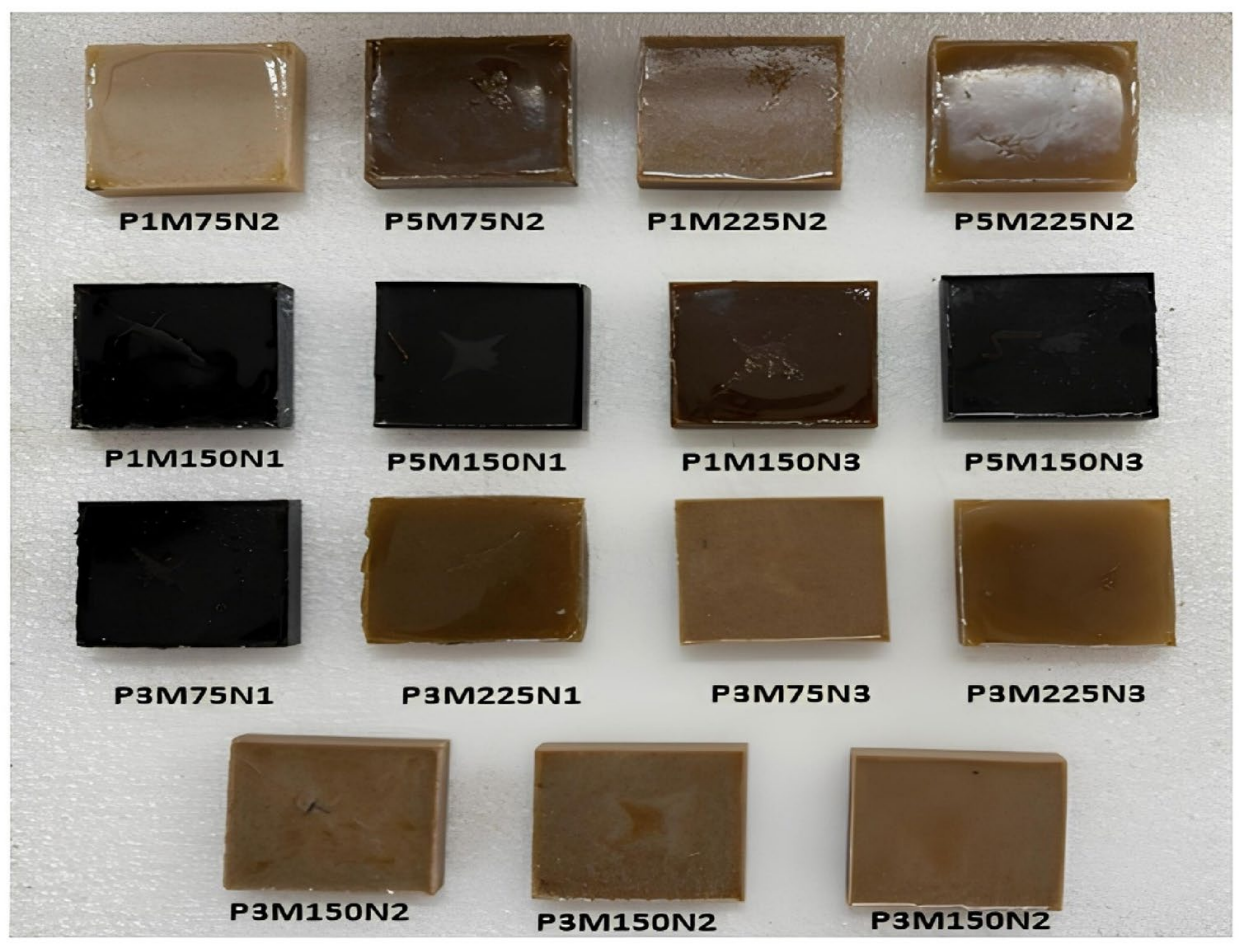
Fabricated epoxy/BFF nanocomposite specimens.

The process of creating the composite material starts with preparing the epoxy resin shown in Fig. 1. First, epoxy is poured into a beaker and then palm fibre is added. This mixture is stirred mechanically for 25 min to ensure everything is evenly distributed. Following this, nanofillers are introduced based on the Box-Behnken Design (BBD) experimental run and the mixture is agitated mechanically for another 25 min to achieve a uniform consistency. Once the mixing is complete, the hardener is added to the resin mixture and stirred manually for 5 min to initiate the curing process. Finally, the resulting slurry is poured into a silicon mould to shape the composite^36^. Figure 2 shows the fabricated samples of epoxy/BFF nanocomposite.

### Theoretical analysis

#### Fiber volume fraction (V_***f***_)

Fiber volume fraction (V_f_), the proportion of fibers in a composite material, is determined based on the rule of mixtures^37^. This parameter is important when it comes to predicting the mechanical properties of the composite material. For composites without nanofillers, V_f_ (Eq. 1) can be calculated as follows,

1 $${V}_{f}= \frac{ \frac{{M}_{p}}{{\rho }_{f}}}{\frac{{M}_{p}}{{\rho }_{p}}+\frac{{M}_{m}}{{\rho }_{m}}}$$

When the nanofillers are added to the composite volume (Eq. 2), another term is introduced to take account of the contribution of the nanofillers to the composite volume; thus, the modification to the equation is:

2 $${V}_{f }= \frac{\frac{{M}_{p}}{{\rho }_{p}}}{\frac{{M}_{p}}{{\rho }_{p}} +\frac{{M}_{m}}{{\rho }_{m}} +\frac{{M}_{n}}{{\rho }_{n}}}$$

where V_f_ is the volume fraction of the composite material, M_p_, M_m_ and M_n_ are the mass of fiber, matrix and nanofillers in grams (g), ρ_f_, ρ_m_ and ρ_n_ are the density of fiber, matrix and nanofillers in g/cm^3^.

The equations assume that fibers and nanofillers are uniformly distributed within the matrix, giving rise to negligible voids. Therefore, the calculated fiber volume fraction shall serve as a platform to evaluate the composition of the material and to compare it to the intended design specification.

#### Experimental density

The water immersion technique can measure the experimental actual density (ρ_exp_) of the composite. The weighing balance is used to measure the mass and the volume of the sample is calculated using the water displacement method before and after the sample immersion. The experimental density is finally given by Eq. (3).

3 $${\rho }_{exp} =\frac{Weight \, of \, the \, Sample (\text{g})}{ Volume \, of \, the \, Sample \left({\text{cm}}^{3}\right)}$$

#### Void content of composites

The Void content (V_cn_) in terms of composite volume percent is calculated using Eq. (4), following ASTM D2734. The theoretical density is calculated using Eq. (5).

4 $${V}_{cn }= 100 \left(\frac{{\rho }_{th }- {\rho }_{exp}}{{\rho }_{th}}\right)$$

5 $${\rho }_{th}= {\rho }_{f }{V}_{f}+{\rho }_{m }{V}_{m} +{\rho }_{n }{V}_{n}$$

where V_cn_ is void content of the composite material, V_f_, V_m_ and V_n_ are volume fraction, matrix and nanofiller.

### Microstructural analysis

The BFF microstructure and voids in selected composite samples are analyzed using an Olympus BX61 optical microscope which generates high-definition images for studying fiber distribution and void morphology.

### Scanning electron microscopy analysis

A Zeiss Evo10 High resolution scanning electron microscope (HR- SEM) with a 10 kV accelerating voltage is used to analyse the morphology of the prepared composite. Before inspection, the surface is sputter-coated with a thin layer of gold. Further, the SEM images are used to measure the quantitative analysis of void content and particle distribution using ImageJ software.

### Impact test

The impact test is performed using J Tech Instruments impact testing machine. The samples are fabricated according to ASTM D256 (75 × 10 × 10 mm^3^) standards. The strength testing is to be conducted following Izod testing principle.

### Hybrid optimization methodology

**Fig. 3:**
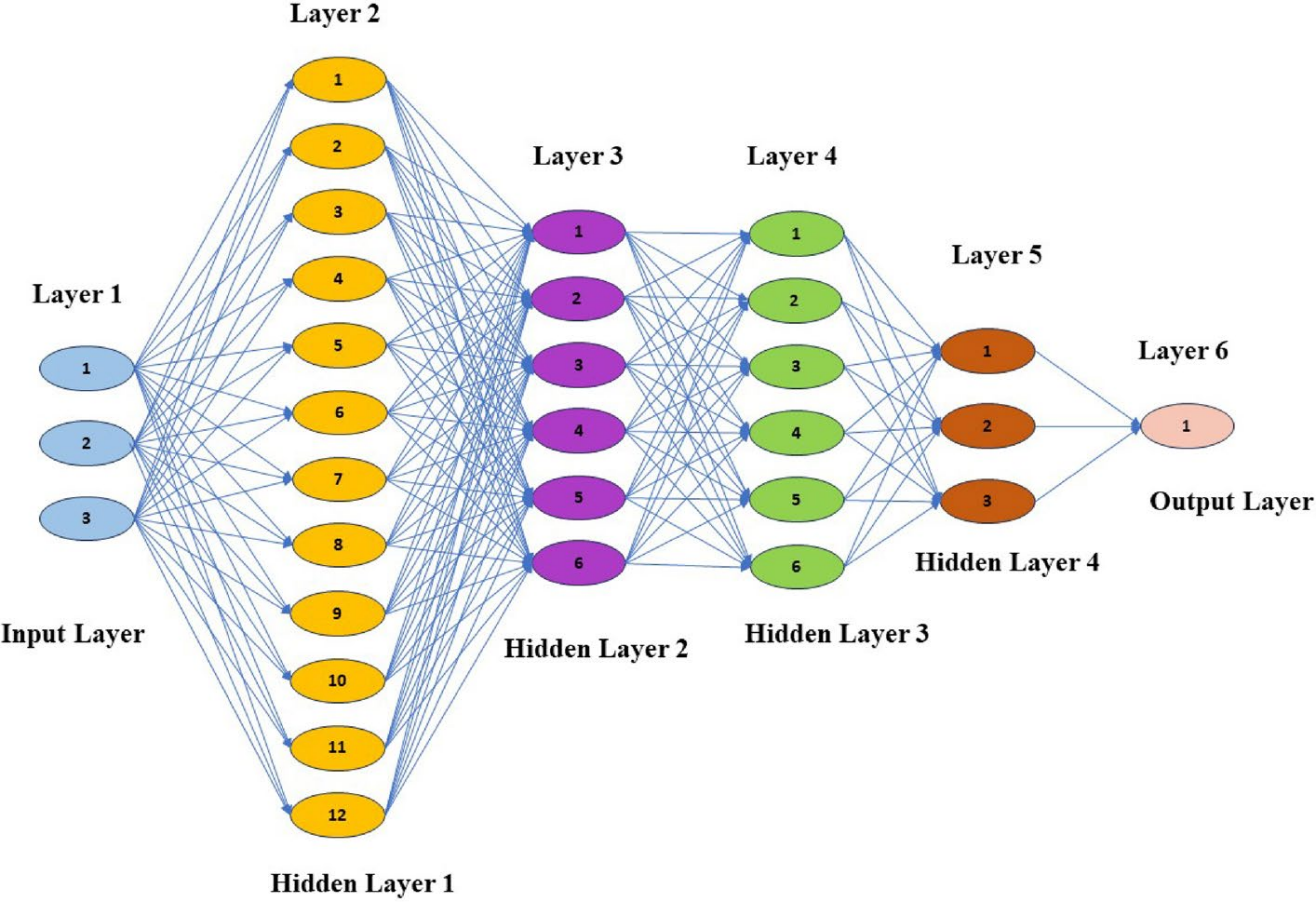
ANN architecture for void content.

**Fig. 4:**
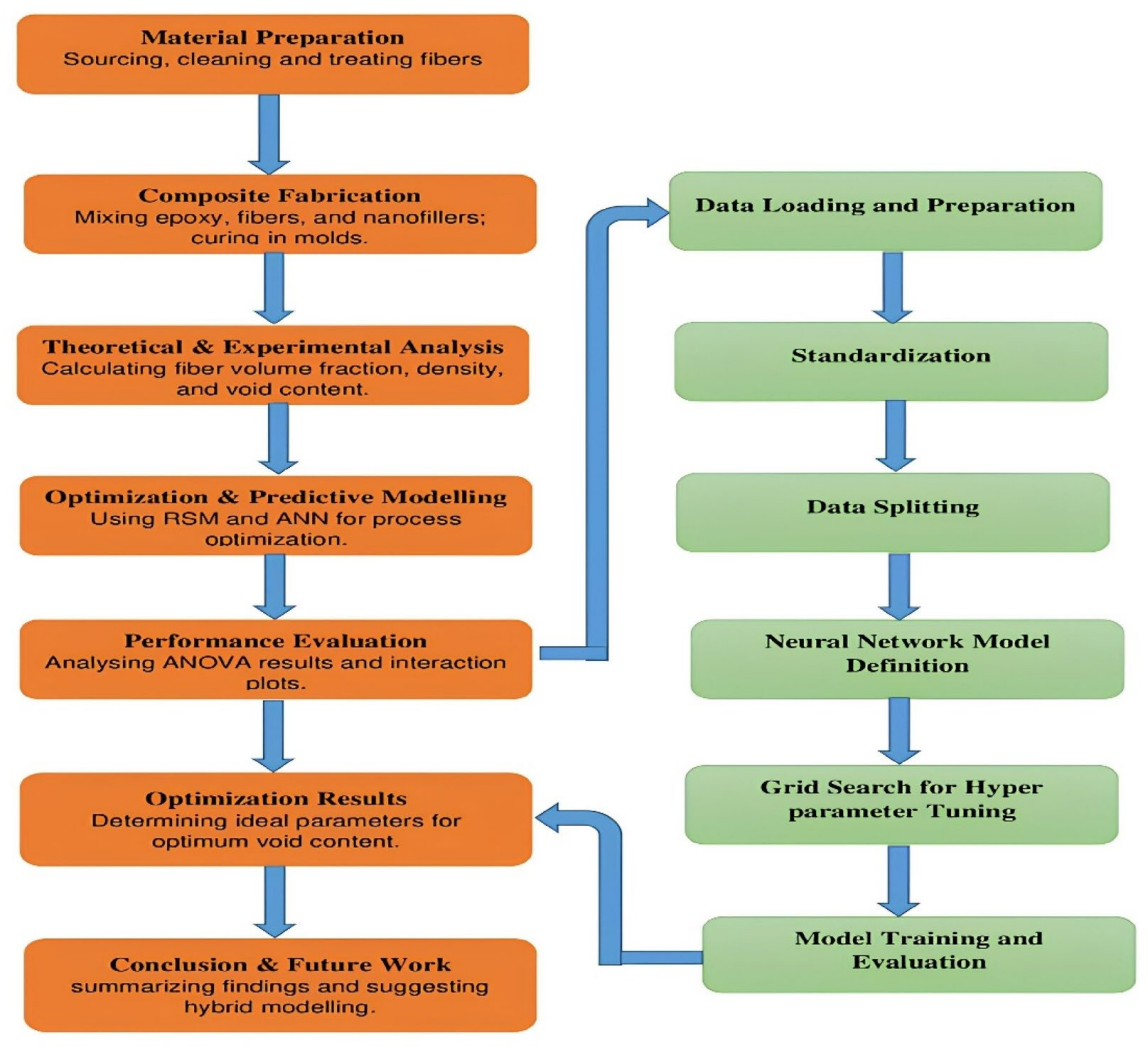
Methodology of the study.

The parameters studied in this investigation are BFF weight percentage (wt%), BFF mesh size (µm) and nanofillers (MWCNT, h-BN and Al_2_O_3_) as shown in Table 1. Response Surface Methodology (RSM) with a Box-Behnken Design (BBD) approach achieves optimal void content. Moreover, the artificial neural network (ANN) is employed to further improve the optimization process. For added certainty, synthetic data-generating algorithms are exploited, yielding more data points that would fine-tune the predictive strength of our model^24–26^. Figure 3 illustrates the ANN architecture and Fig. 4 mentions the methodology flow diagram of this study.

**Table 1:** Prepared bio-nanocomposite combination done through the Design of Experiment.

Variable	Parameter		Level		
	Factor	Unit	− 1	0	1
P	BFF weight content	%	1	3	5
M	BFF mesh size	µm	75	150	225
N	Nanofiller	%	MWCNT(1)	h-BN(2)	Al_2_O_3_ (3)

The experimental work studied the effect of fiber content, mesh size and type of nanofiller on void content. This produced an extensive data set through Box-Behnken Design. In another step, artificial neural network modelling was implemented to be able to predict the values of void content based on the data collected from the experimental results to be able to design the composite into a better one.

## Results and discussions

### Fiber volume fraction determination

Table 2 shows the fibers and nanofillers volume fraction of the BFF-reinforced nanocomposites as determined by utilizing the easy, non-destructive method and applying the rule of mixtures as outlined by Eqs. (1) and (2). These fibre volume fractions, additional nanoparticles and known densities of fiber and nanofiller are applied to compute the observed density.

**Table 2:** The fiber volume fraction of prepared bio-nanocomposite.

Sample name	Fiber volume fraction	Fiber mesh size	Nanofiller volume fraction
P1M75N2	1.6403	75	0.5103
P5M75N2	7.9964	75	0.4976
P1M225N2	0.9634	225	0.5138
P5M225N2	4.8253	225	0.5147
P1M150N1	1.2800	150	0.5486
P5M150N1	6.3296	150	0.5425
P1M150N3	1.2833	150	0.2924
P5M150N3	6.3457	150	0.2892
P3M75N1	4.8569	75	0.5397
P3M225N1	2.8917	225	0.5508
P3M75N3	4.8692	75	0.2876
P3M225N3	2.8992	225	0.2936
P3M150N2	3.8202	150	0.5094
P3M150N2	3.8202	150	0.5094
P3M150N2	3.8202	150	0.5094

Volume fraction of fiber is affected by nanofillers types used, mesh size and percentage of fiber weight sewn controlled attributes, like better packing, which makes the mesh size smaller by increasing fiber weight. The MWCNT, placed at low density and dispersed well across the fiber, is the peak contributor to fiber percentage enhancement, whereas h-BN contributes a little less than MWCNT and Al_2_O_3_ gets the lowest, as it gets embedded with a high weight-to-volume ratio.

### Experimental and theoretical densities

**Fig. 5:**
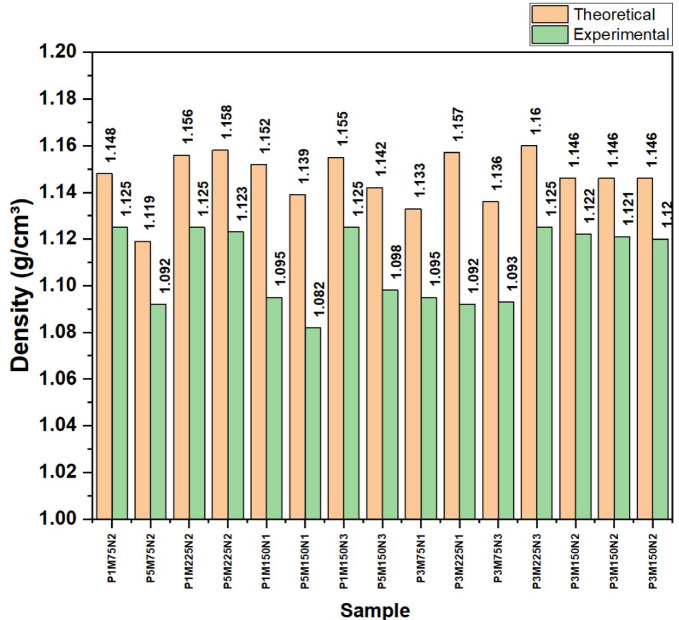
The comparison between theoretical and experimental densities.

The experimental & theoretical densities (Fig. 5) of composite samples are profoundly influenced by factors like fiber weight percentage, fiber mesh size and the type of nanofiller. Due to incomplete matrix infiltration, higher fiber content often leads to enlarged porosity^38^ and lower experimental density. Fiber mesh size affects packing efficiency^39^, with finer fibers improving matrix interaction and reducing voids, while coarser fibers may introduce inconsistencies. The type of nanofiller also plays a role, as materials like MWCNT and h-BN enhance packing efficiency, whereas Al_2_O_3_ may lead to agglomeration, affecting density distribution. The deviations between theoretical and experimental densities bring into light the requirement of optimizing the processing conditions for better composite quality.

### Void content

**Fig. 6:**
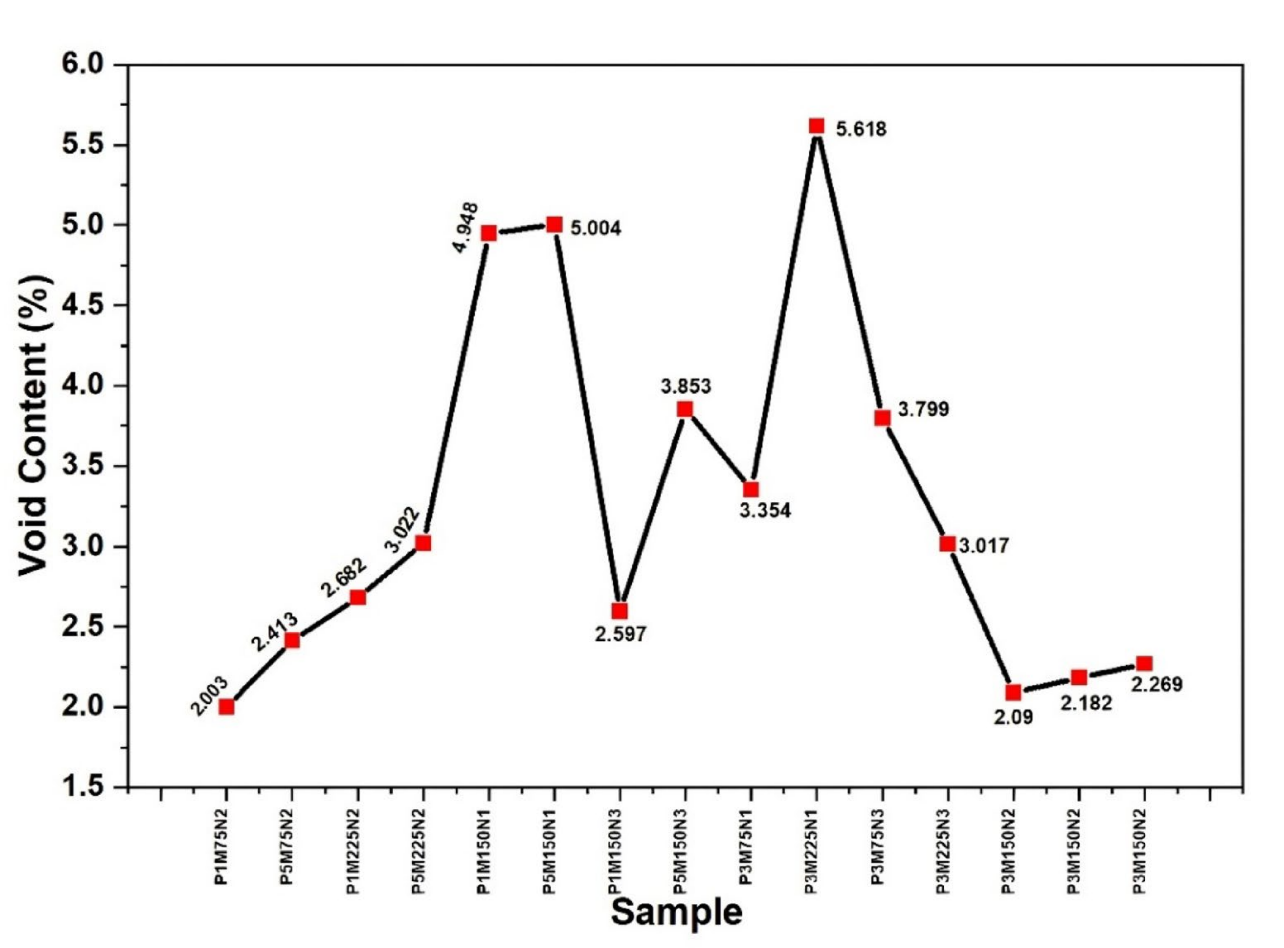
Void content of prepared bio-nanocomposites.

The void content of the produced bio-nanocomposites, as presented in Fig. 6, is varied depending on sample composition, which is indicative of effects due to fibre concentration, fibre content and filler combinations. The results show that void content is affected by the compatibility of the bio based fibres and epoxy matrix and the dispersion efficiency of ceramic and nanofiller. Samples with a higher fibre content and filler loading like P3M150N1 and P3M225N1 have an increased void content (5.004 and 5.618%, respectively) and this could be attributed to greater fibre-matrix interfacial gap. Conversely, samples with lower fibre and filler content like P1M75N2 and P3M150N2 exhibit lower void content (2.003 and 2.090%, respectively) owing to improved matrix infiltration with lesser air entrapment. This variation in void content for the various formulations brings to the fore the need to optimize fiber-to-filler ratios to minimize the voids and harness the performance of the composite, especially in applications that require improved dielectric and thermal properties.

### Microstructural analysis and particle characteristics

**Fig. 7:**
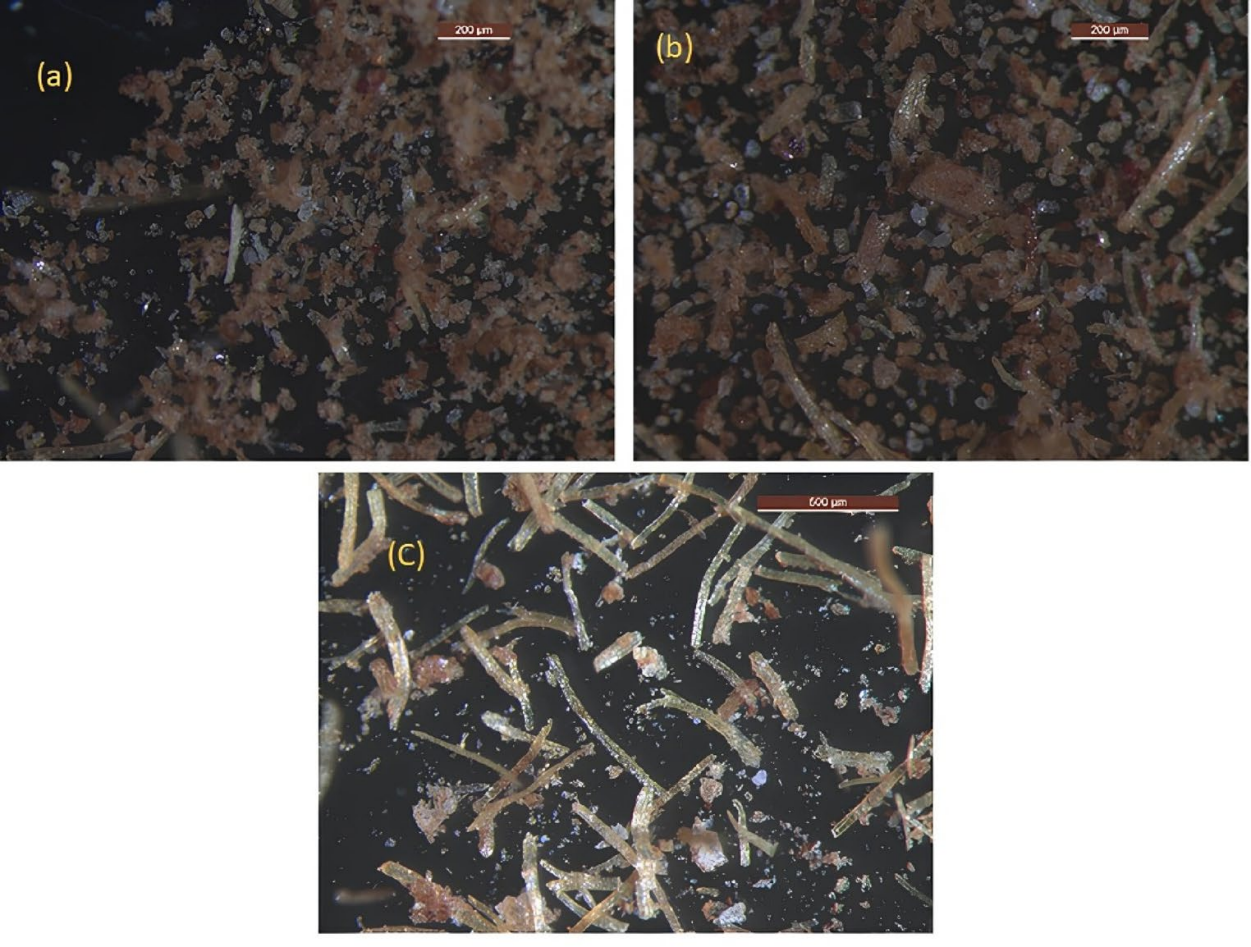
BFF microstructure (**a**) 75 µm (**b**) 150 µm (**c**) 225 µm.

**Fig. 8:**
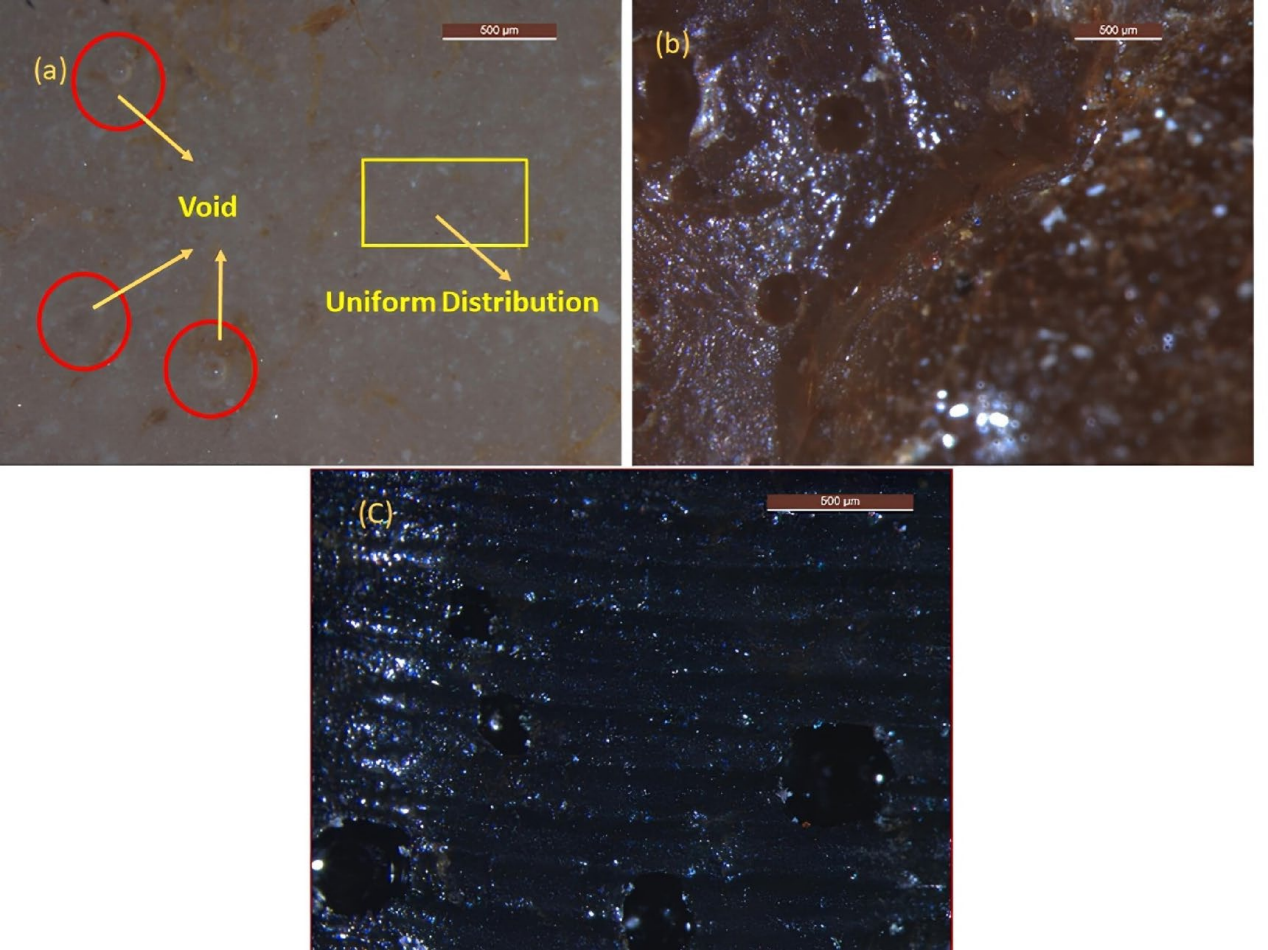
Void microstructure of epoxy/BFF nanocomposite (**a**) P1M75N2 (**b**) P5M150N3 (**c**) P3M225N1.

The microstructure of BFF and its epoxy nanocomposites provides critical insights into void content and material behaviour. Particle sizes ranging from 75 to 225 µm are carried out using manual sieving after crushing. Figure 7: BFF Microstructure (a, b, c) shows fibers with 75, 150 and 225 µm, respectively, with elongated, irregular fibers and fragmented, angular particles. Smaller sizes (75 µm) show dense fiber packing, while larger sizes (225 µm) exhibit longer fibers with increased particle complexity, which governs matrix infiltration. Figure 8: Void Microstructure of Epoxy/BFF Nanocomposite (a, b, c) shows void distribution wherein (a) 75 µm samples show evident voids and uniform nanofiller distribution, (b) 150 µm samples demonstrate void irregular patterns which imply agglomeration and (c) 225 µm samples reveal very few voids, suggesting that resin penetration into the composite is enhanced. These observations ascertain the definition of fiber shape (elongated) as well as that of particle shape (angular) supporting the desire of this study to minimize voids.

### SEM analysis

**Fig. 9:**
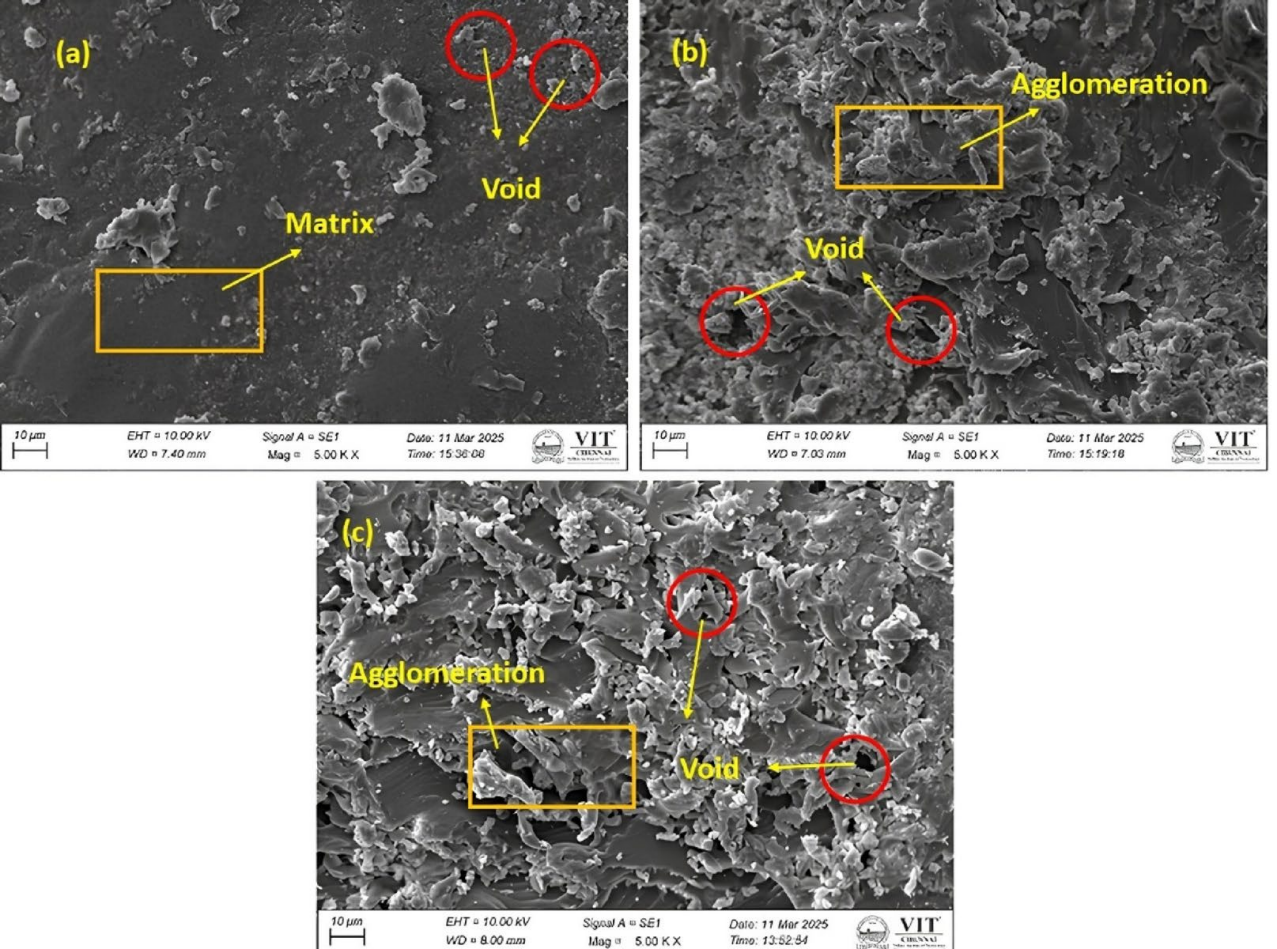
SEM analysis of composite material (**a**) P1M75N2 (**b**) P5M150N3 (**c**) P3M225N1.

The SEM micrographs of bio-composites with hBN, Al_2_O_3_ and MWCNT nanofillers at 1% concentration reveal distinct morphological characteristics influencing void formation as shown in Fig. 9. Samples P1M75N2, P5M150N3 and P3M225N1 were selected to represent the range of void content (2.00348–5.61798%) and parameter variations in fiber weight, mesh size and type of nanofiller in a 15 sample set, by giving insight into the defect formation across the experimental design. The analysis of the composite surfaces highlights variations in nanofiller dispersion, matrix uniformity and void distribution. Micrographs of hBN reinforced composites exhibit a relatively smooth matrix with minimal voids, suggesting better filler integration and reduced air entrapment. In Al_2_O_3_ reinforced composites, void formation is more noticeable due to partial agglomeration of nanoparticles, leading to microvoids around clustered regions. SEM images of MWCNT-reinforced composites display the highest void content, characterized by significant nanofiller aggregation and irregular void structures. The presence of MWCNT entanglement and particle clustering increases the likelihood of air entrapment, resulting in more pronounced voids compared to hBN and Al_2_O_3_. The *Borassus flabellifer* fiber used in the composite has a micron size of 75 µm, contributing to the overall structural integrity by providing reinforcement. The micrographs confirm that void formation intensifies with increasing nanofiller agglomeration, affecting the homogeneity of the composite structure.

#### Quantitative analysis from SEM image

##### Void content analysis

**Fig. 10:**
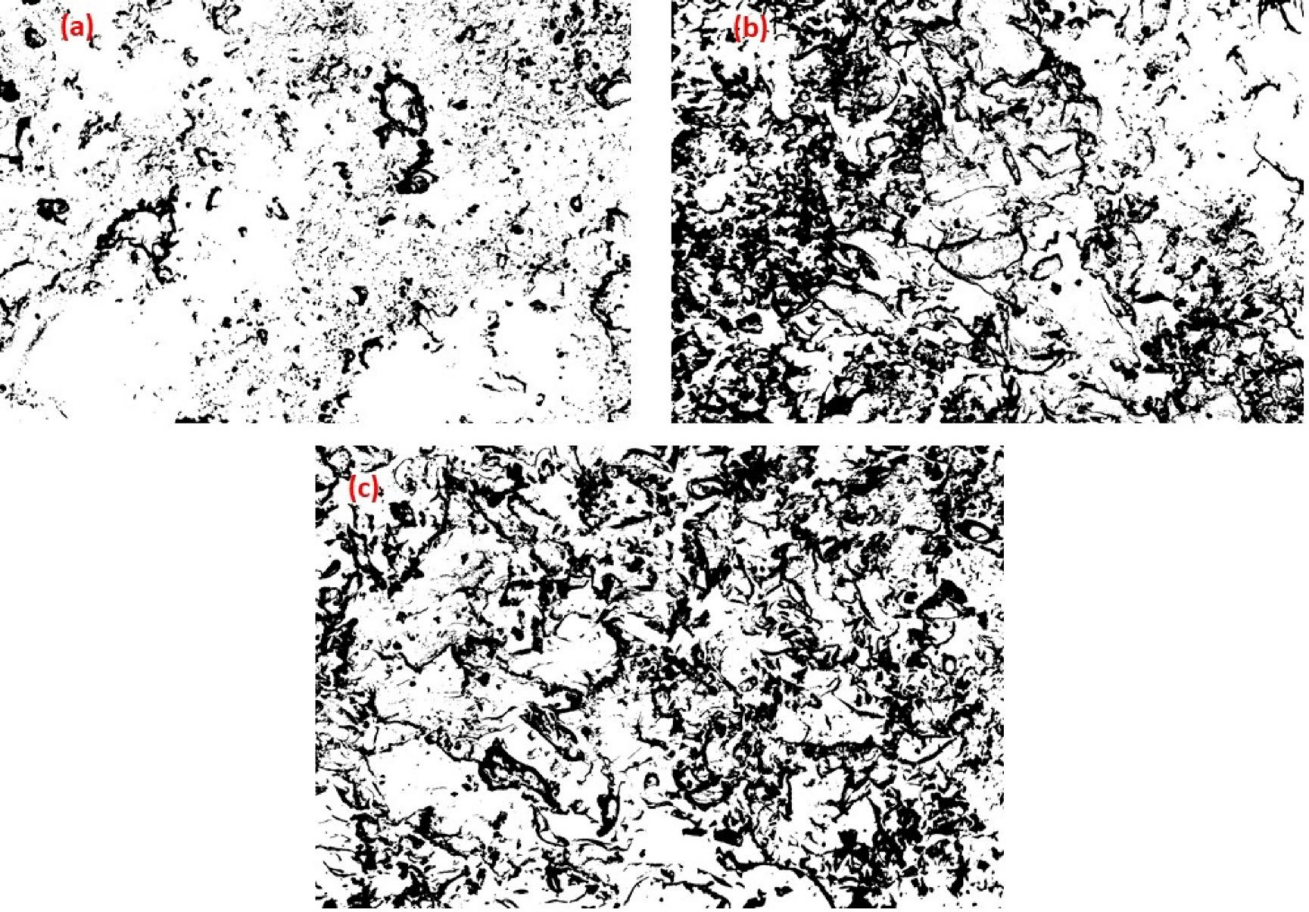
Void content analysis by SEM images (**a**) P1M75N2 (**b**) P5M150N3 (**c**) P3M225N1.

SEM micrographs in Fig. 8 and statistics in Table 3 disclose differentiated void content trend in three samples: Fig. 10(a) P1M75N2, (b) P5M150N3 and (c) P3M225N1. P1M75N2 demonstrates small, evenly spaced voids, suggesting a dense structure because of low fiber content and small mesh size, with Nano hBN facilitating even packing. P5M150N3 has moderate, unevenly spaced voids, indicating decreased density from increased fiber content and increased mesh size, with Nano alumina facilitating gap creation. P3M225N1 shows the greatest void content with large, dispersed clusters, stimulated by coarser mesh size and possible agglomeration from Nano MWCNT, creating a porous structure.

**Table 3:** Quantitative analysis of void content detection from SEM images.

Sample	Count	Total area	Avg. size (µm^2^)	%Area
P1M75N2	96	29,415	306.406	1.045
P5M150N3	539	110,640	205.269	3.935
P3M225N1	650	182,180	280.277	6.46

##### Particle distribution analysis

**Fig. 11:**
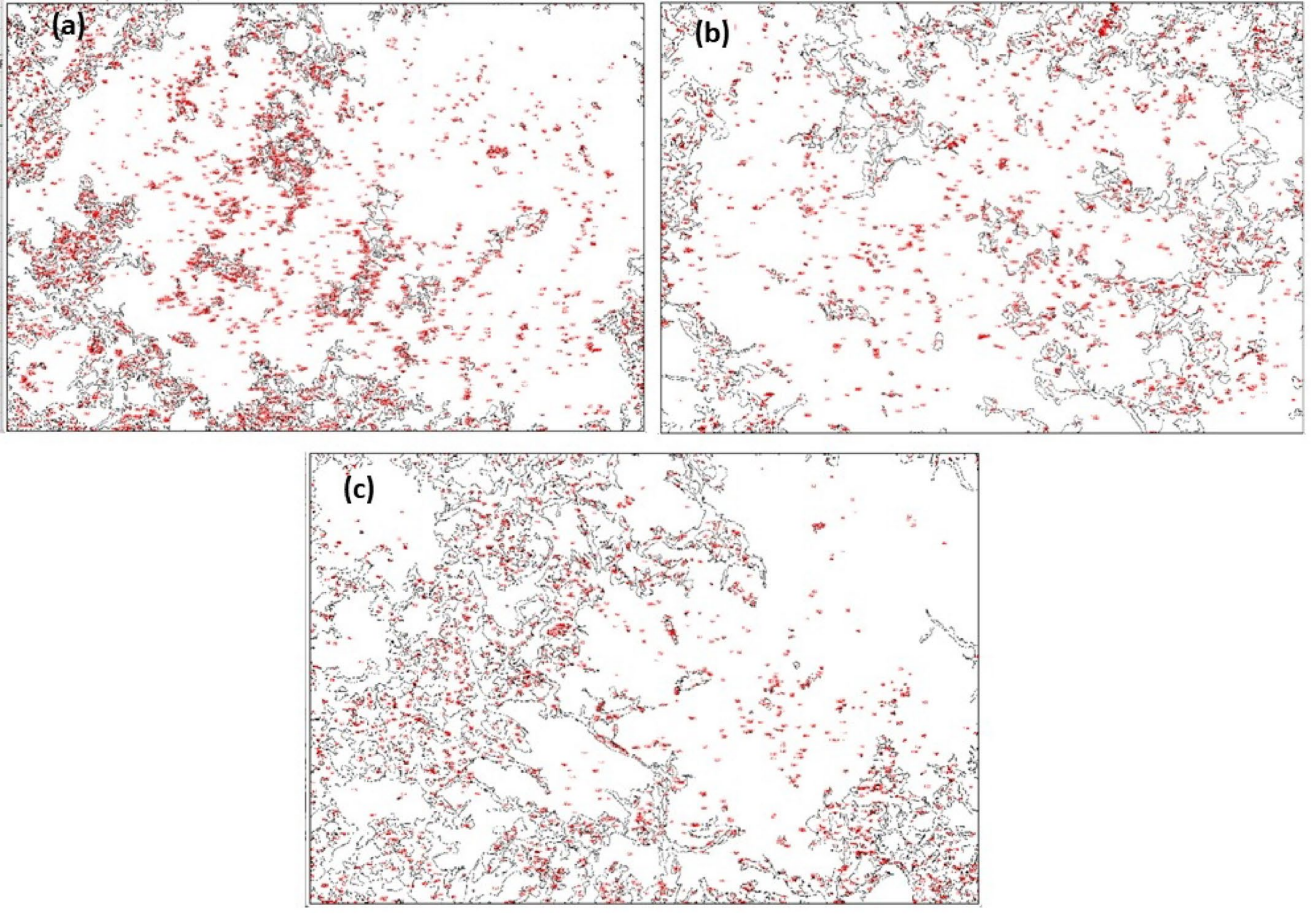
Particle distribution analysis by SEM images (**a**) P1M75N2 (**b**) P5M150N3 (**c**) P3M225N1.

The SEM photographs in Fig. 11, supplemented by the data in Table 4, present clear particle distribution and morphology features in three samples: Fig. 9(a) P1M75N2, (b) P5M150N3 and (c) P3M225N1. P1M75N2 exhibits a very even and well-dispersed particle arrangement, which is due to the low 1% fiber content and narrow 75 micron mesh size, while nano-hBN improves dispersion by minimising particle agglomeration. P5M150N3 shows a moderately dispersed arrangement, with many different particle shapes and visible clustering, which is due to the increased 5% fiber content and wider 150 micron mesh size, while nano alumina slows but does not stop uniformity. P3M225N1 exhibits significant agglomeration with densely packed clusters of large particles, due to the coarser 225 micron mesh size and the high aspect ratio of nano MWCNT, which promotes complex network formation and inadequate dispersion.

**Table 4:** Quantitative analysis of particle distribution and morphology from SEM images.

Sample	Count	Total area	Avg. size (µm^2^)	%Area	Circ	Solidity	Feret diameter (µm)	Verdict
P1M75N2	3142	1,913,198	608.911	68.039	0.801	0.852	8.773	Highly uniform and dispersed
P5M150N3	2064	1,827,335	885.337	64.986	0.826	0.876	8.485	Moderate dispersion
P3M225N1	1913	1,836,861	960.199	65.135	0.784	0.852	12.574	Agglomerated, poor dispersion

### Impact Strength

**Fig. 12:**
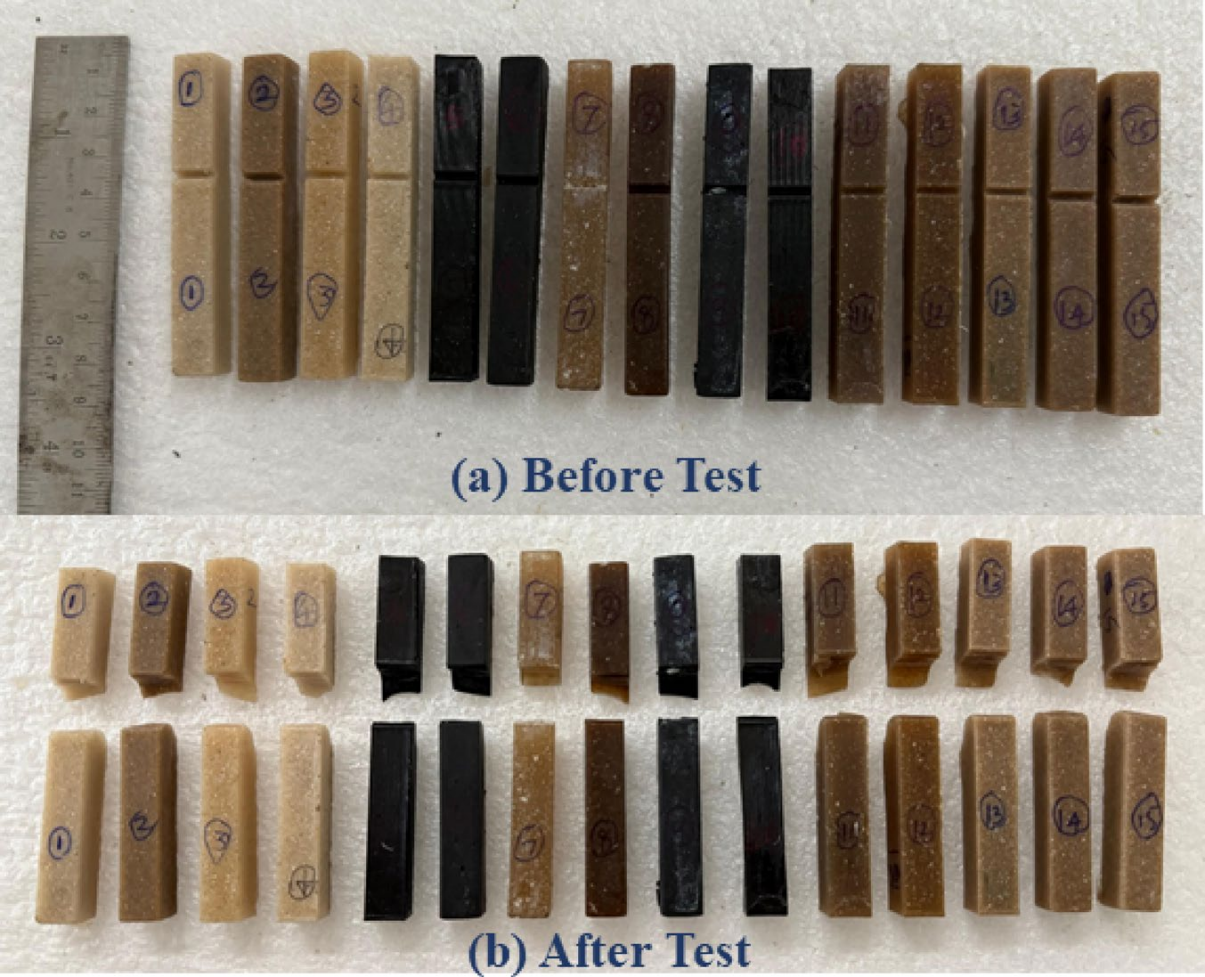
Impact test specimen.

**Fig. 13:**
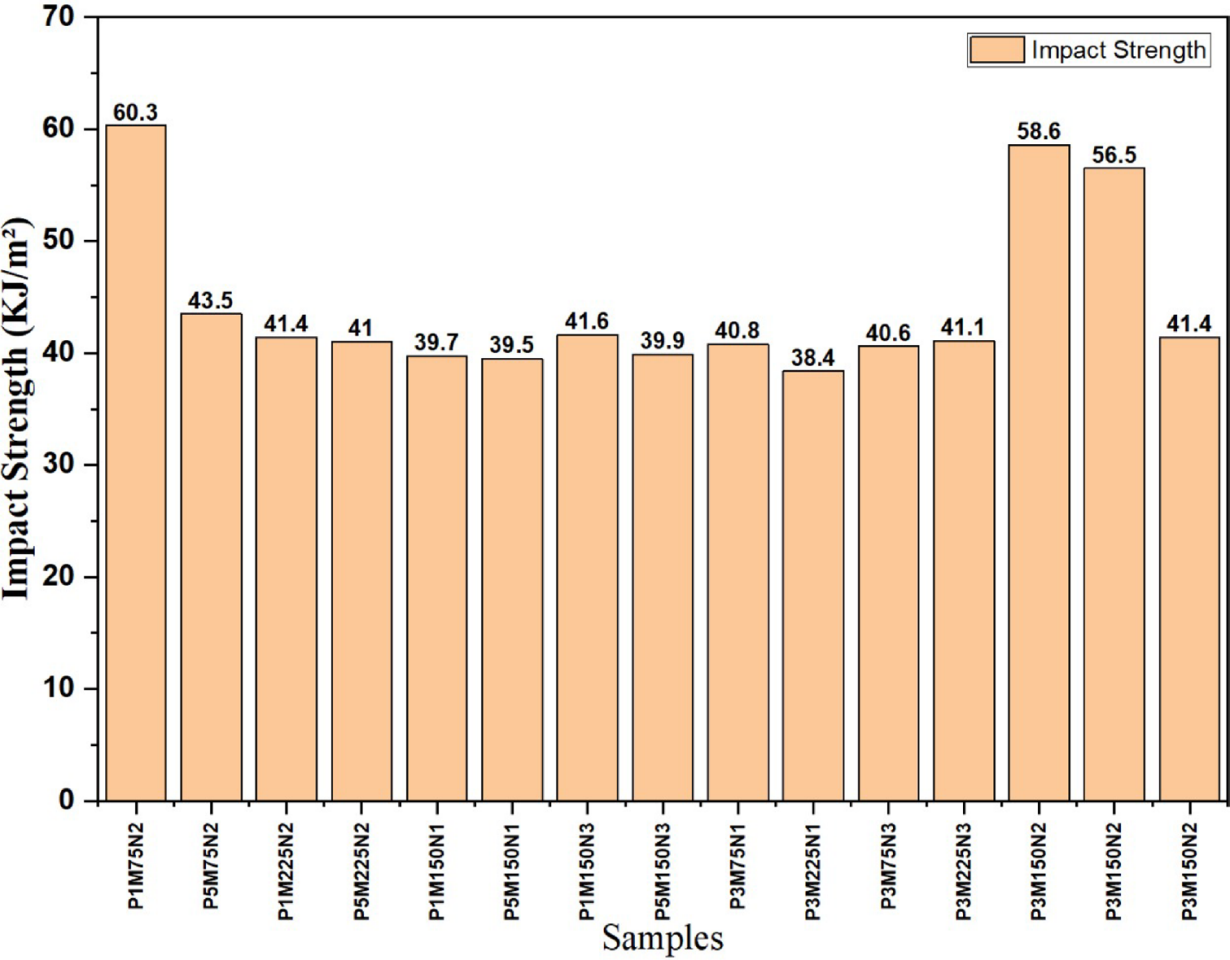
Impact strength of bio epoxy nanocomposite.

Figure 12 shows the before and after testing of the impact test specimen. The bar chart representing impact strength through epoxy/BFF nano composite samples, ranging between 38.4 kJ/cm^2^ (P3M225N1) and 60.3 kJ/cm^2^ (P5M75N2), highlighted influences of material parameters and void content on toughness as shown in Fig. 13. The research has shown that void content, which ranged from 2.00348 to 5.61798%, negatively affects impact strength due to stress concentration points that initiate cracks, reducing the composite ability to absorb energy before failure^40^. Higher void contents, for example, that of P3M225N1, reaching 5.61798% (with an impact strength of 38.4 kJ/m^2^), could plausibly be attributed to poor dispersion of nanofillers or insufficient bonding of resins with the coarser fibers and this weakens the interfacial bonding while enhancing crack propagation. Lower void contents like P1M75N2, with values of 2.00348% and 43.5 kJ/m^2^ and more optimally exploiting h-BN (P5M75N2 at 60.3 kJ/m^2^), could lead to a much higher impact strength via better load transfer and integrity of matrix; Erasing voids achieved by optimizing fiber weight, mesh size and nanofiller selection could thus be established as crucial in relative importance.

### Design of experiment

An adequately constructed study provides valuable data regarding the impact on response variable. This study uses fiber weight percentage, fiber mesh size and nanofiller type as input parameters to void content. BBD design is used to test each parameter at three different levels and the interactions in BFF fibre-reinforced nanocomposites are investigated in detail. Trials are carried out with the 15 samples, changing process parameters like the fiber wt% (1, 3 and 5%), Fiber Mesh size (75, 150 and 225 µm) and type of nanofiller wt% (MWCNT, h-BN and Al_2_O_3_)^41,42^. The study assesses void content and uses the experimental methodology given in Table 1 to discover the optimal void content features of fibre-reinforced epoxy nanocomposites.

The Box-Behnken Design (BBD) with 15 experimental runs was selected for its efficiency in evaluating the effects of fiber weight percentage, mesh size and nanofiller type on void content while minimizing the number of trials. This design provides adequate statistical power (approximately 80%) to detect significant main effects and interactions, assuming moderate effect sizes (f ≈ 0.35), as calculated using standard power analysis for a three-factor BBD. However, the limited sample size may reduce sensitivity to small experimental variations, such as inconsistencies in fiber dispersion or matrix curing, potentially underestimating variability. While the current design yielded consistent trends (e.g., void content ranging from 2.003 to 5.618%), additional replicates beyond the center point repeats (3 runs) could enhance precision and confirm reproducibility.

### ANN model performance

#### Artificial neural network

An Artificial Neural Network (ANN) is developed to predict void content in epoxy-based *Borassus flabellifer* fiber (BFF) composites by modeling relationships between processing parameters: nanofiller type, fiber mesh size and fiber weight percentage. Using a supervised learning approach with backpropagation, the ANN is trained on experimental data to minimize prediction errors. Hyperparameter optimization in Python fine-tunes the model’s architecture (e.g., number of layers, neurons, activation functions and learning rate) to enhance accuracy and generalization. The trained ANN reliably predicts void content, identifying parameter combinations that minimize voids, thus reducing the need for extensive experimental trials. This predictive tool enhances understanding of void formation and supports efficient bio-nano composite design.

#### Model architecture

The ANN is a multi-layer feed-forward network designed for regression, predicting continuous void content (Fig. 3). The input layer accepts five features: fiber weight percentage, fiber mesh size (numerical) and nanofiller type (one-hot encoded into three categorical variables). The architecture includes four hidden layers:

- First layer: 12 neurons with ReLU activation to capture non-linear patterns.
- Second and third layers: 6 neurons each with ReLU, refining feature representations and aiding regularization.
- Fourth layer: 3 neurons with ReLU, further distilling features. The output layer has one neuron with linear activation for continuous porosity predictions. This layered design balances expressiveness and generalization, preventing overfitting. The model is compiled using the Adam optimizer, with Mean Absolute Error (MAE) as the loss metric, providing interpretable error in void content percentage units.

#### Model training and validation

**Fig. 14:**
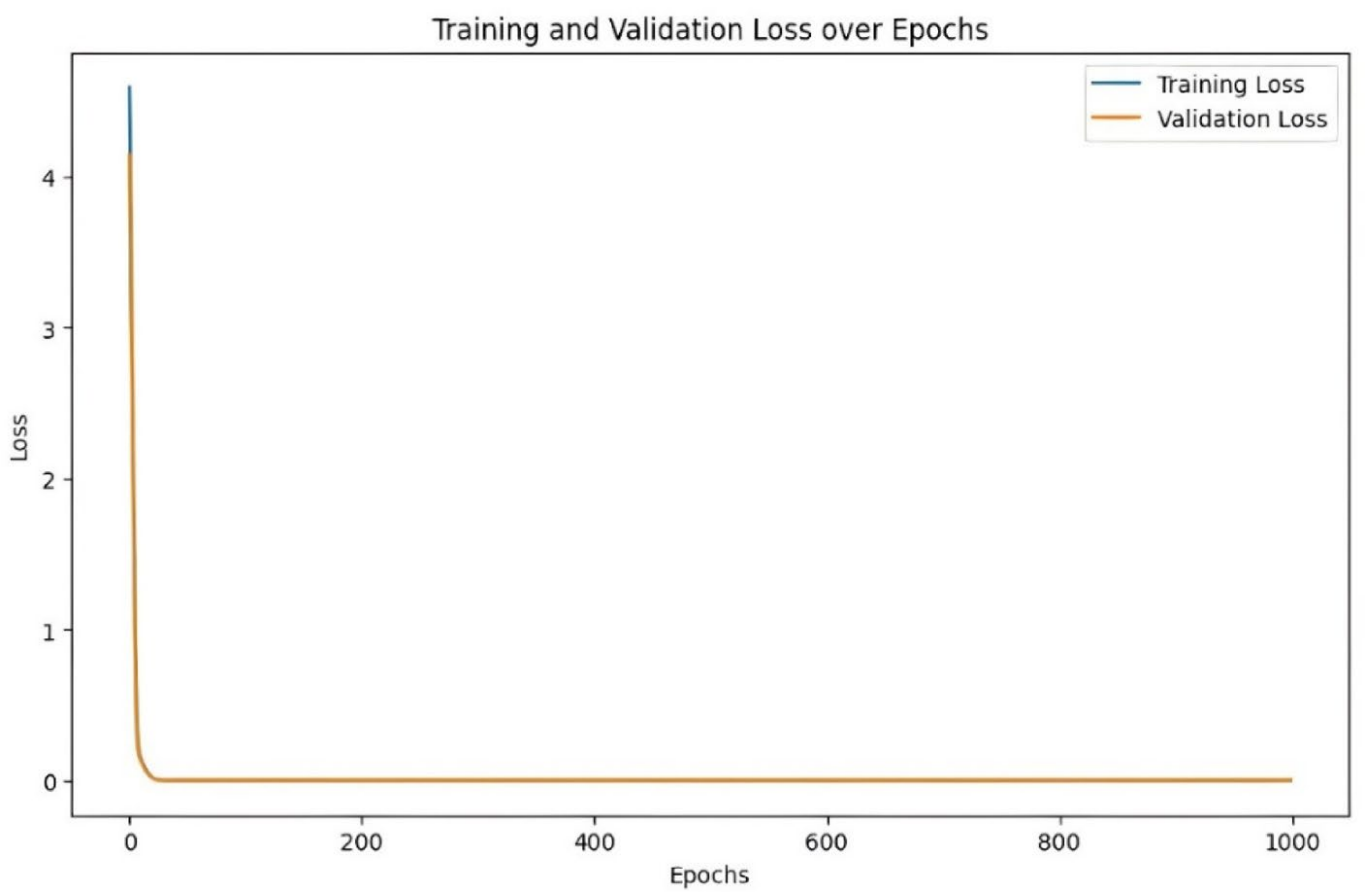
Training and validation loss performance of porosity model.

**Fig. 15:**
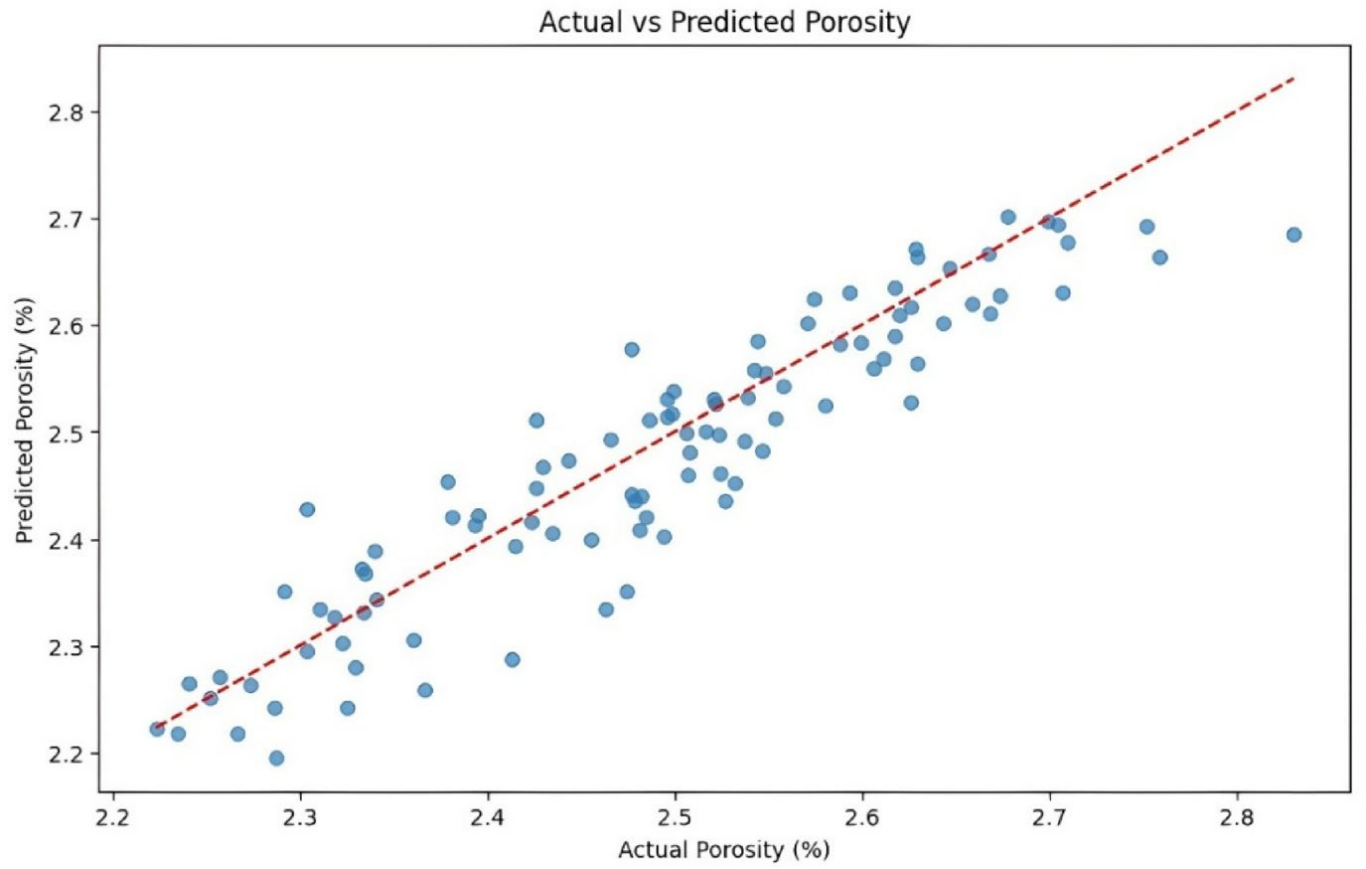
ANN performance of porosity model.

The dataset is split 80:20 into training and validation sets and the model is trained for 1000 epochs. Training adjusts weights to minimize loss, while validation monitors generalization. Training and validation losses are plotted (Fig. 14) to assess performance. Consistent or decreasing validation loss indicates good generalization, while diverging losses suggest overfitting. The plots confirm stable learning without significant overfitting. A scatter plot of observed versus predicted values (Fig. 15) shows tight clustering along the diagonal, confirming reliable predictions with minimal deviation. These results highlight the ANN’s precision and robustness for void content prediction.

#### K-fold cross validation in the program

The present program, K-Fold Cross Validation, is applied to measure the performance and generalizability of the artificial neural network (ANN) model. The dataset in this case is divided into five folds such that each is held out as a test set about once while the remaining comprise the training set. For every fold, the model is trained on the training subset and evaluated on the test subset for the Mean Absolute Error (MAE) and R-squared (R^2^) metrics upon each evaluation as shown in Table 5. This iterative approach helps lessen the risks of model overfitting, while at the same time ensuring the model performance is not reliant on an arbitrary train-test split. After completing these five-folds, the program computes the average MAE and R^2^ scores, thus giving a stronger assurance of the good model performs in prediction. This method ensures that the model is exposed to different subsets of the data, thereby making it dependable in real-life scenarios.

**Table 5:** K-fold cross-validation of machine learning models.

Model	Initial value		Final value after K fold validation (K = 5)	
	MAE	R^2^	MAE	R^2^
Void content	0.0461	0.9470	0.0400	0.9583

### Regression model

The experimental data are analysed systematically with Design Expert 13, being among the most contemporary statistical applications dedicated partly to determine the essential coefficients required in a delicate mathematical model. Judiciously selected process variables are utilized for brilliantly managing the optimization of void contents. Quadratic models for void content are then chosen after carefully weighing the statistical significance against the extreme proximity of adjusted and predicted R^2^ values. This extremely close connection illustrates that effective quadratic models describe the sophisticated relationship between process data and its associated factors. The strange machined performances are revealed in the form of Eq. (6) in the case of BFF-reinforced epoxy nanocomposites, as they encapsulate these enigmas. These three parameters are of utmost importance- fiber wt%, fiber mesh size and type of nanofiller as they determine the outcome. An extensively established coefficient of correlation (R^2^) serves as a guide toward better forecasting. These plots underline the very close fit of the regression lines with the actual outcomes. The R^2^ values from void content models indicate a fair level of understanding and mastery, taking 0.9427.

6 $$\begin{aligned} {\mathbf{Void}} \, {\mathbf{Content}} & = { 7}.{95159 } - \, 0.{531385 }*{\text{ A }} + \, 0.0{2}00{273 }*{\text{ B }} - { 6}.{3}0{883 }*{\text{ C }} - \, 0.000{114321 }*{\text{ AB}} \\ & \quad + \, 0.{149877 }*{\text{ AC }} - \, 0.0{1}0{1537 }*{\text{ BC }} + \, 0.0{629439 }*{\text{ A}}^{{2}} + { 1}.{\text{74642e}} - 0{5 }*{\text{ B}}^{{2}} + { 1}.{66877 }*{\text{ C}}^{{2}} \\ \end{aligned}$$

#### Analysis of variance for void content

ANOVA results for void content (Table 6) indicate that the model is highly significant (*p* = 0.0005, F = 35.86), explaining a substantial portion of the variance in void content. Among the factors, nanofiller (C) has the most pronounced effect (*p* = 0.0004, F = 69.74), suggesting its strong influence in reducing void content, likely due to improved resin infiltration and matrix densification. Fibre mesh size (B) and fibre weight (A) also play significant roles, with p-values of 0.0095 and 0.0286, respectively. A finer mesh size may enhance resin wetting, while fibre weight influences consolidation and compaction.

**Table 6:** ANOVA for void content model.

Source	Sum of squares	df	Mean square	F-value	*p*-value	
Model	18.52	9	2.06	35.86	0.0005	Significant
A-Fiber Wt	0.5316	1	0.5316	9.27	0.0286	
B-Fiber mesh size	0.9589	1	0.9589	16.72	0.0095	
C-Nanofiller	4.00	1	4.00	69.74	0.0004	
AB	0.0012	1	0.0012	0.0205	0.8917	
AC	0.3594	1	0.3594	6.27	0.0543	
BC	2.32	1	2.32	40.44	0.0014	
A^2^	0.2341	1	0.2341	4.08	0.0994	
B^2^	0.0356	1	0.0356	0.6212	0.4663	
C^2^	10.28	1	10.28	179.25	< 0.0001	
Residual	0.2868	5	0.0574			
Lack of fit	0.2708	3	0.0903	11.31	0.0824	Not significant
Pure error	0.0160	2	0.0080			
Cor total	18.80	14				

The significant interaction between fibre mesh size and nanofiller (BC, *p* = 0.0014) indicates a combined effect, implying that nanofiller performance may be dependent on fibre distribution and permeability. In contrast, interactions AB and AC are not significant, suggesting that fibre weight does not interact strongly with other factors. The quadratic effect of nanofiller (C2) is highly significant (*p* < 0.0001), confirming that excessive nanofiller may lead to agglomeration, counteracting its benefits. However, the quadratic terms A2 and B2 are not significant, indicating a more linear relationship for fibre weight and mesh size. The non-significant lack of fit (*p* = 0.0824) suggests a good model fit. Overall, nanofiller is the dominant factor in controlling void content, followed by fibre weight and mesh size. These findings highlight the importance of optimizing nanofiller content and fibre parameters to minimize voids and enhance composite quality.

**Fig. 16:**
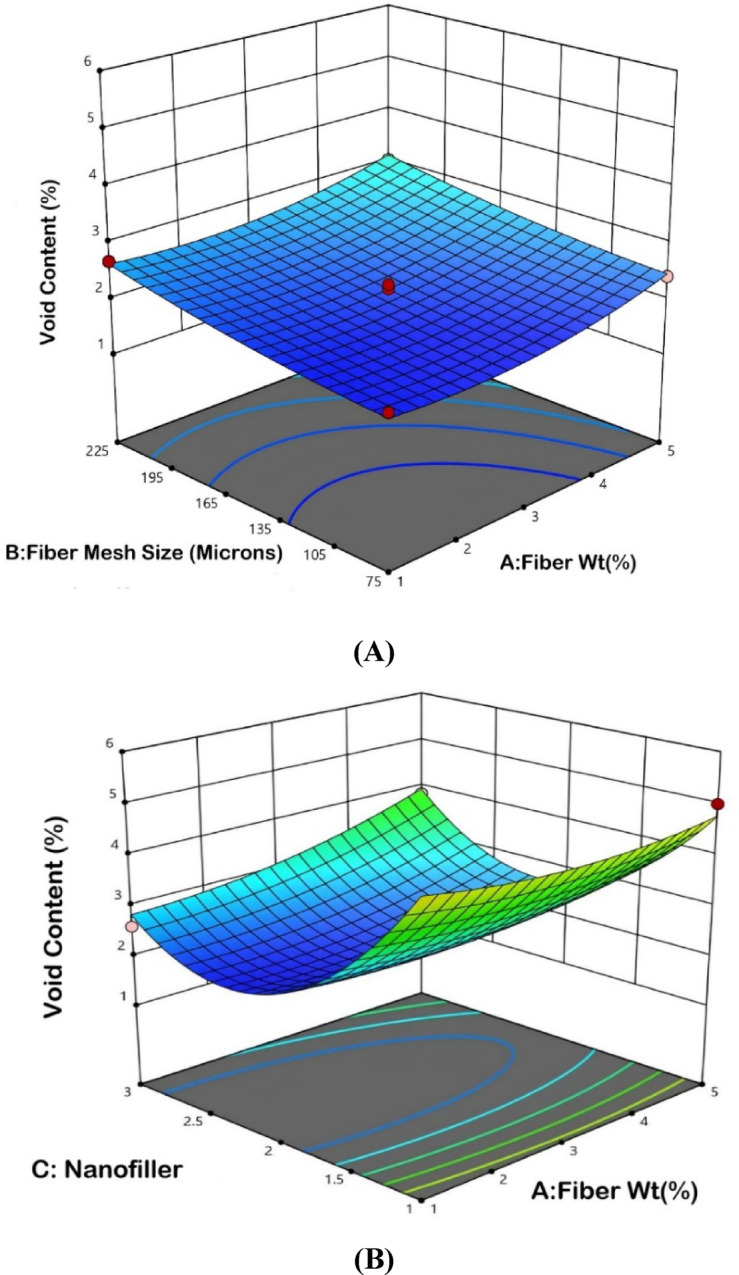

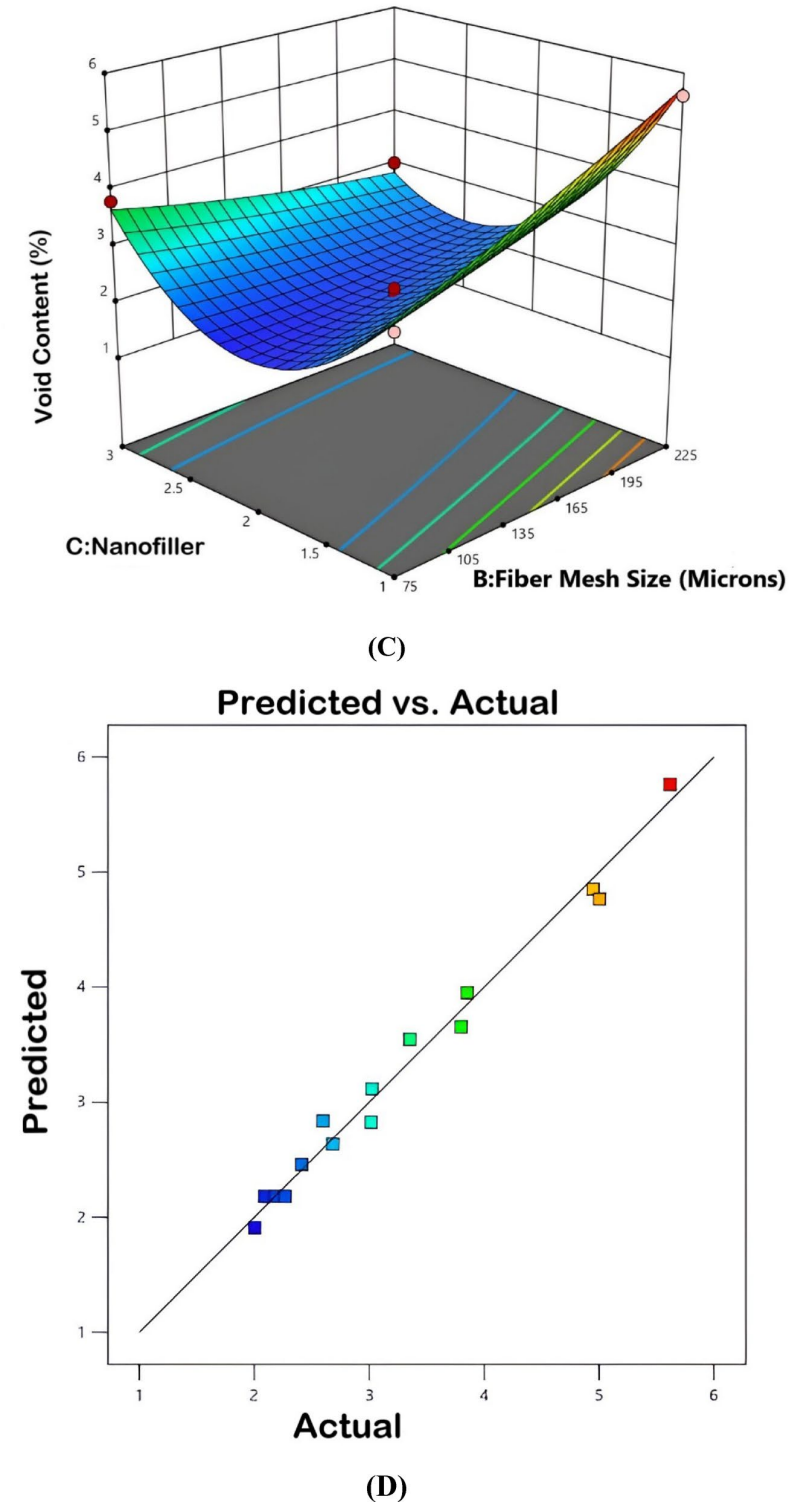
Interaction plot for void content.

The interaction plots in Fig. 16 display the combined effects of fiber weight, fiber mesh size and varying types of nanofillers-MWCNT, h-BN and Al_2_O_3_-on the void content of bio-nanocomposites. Plot (A) shows the interaction between fiber weight and fiber mesh size, whereby void content increases with increasing fiber weight, whereas mesh size has much less influence. In plot (B), the interaction between fiber weight and nanofiller shows that the void content increases considerably with increasing fiber weight and concentration of nanofiller, especially in the case of Al_2_O_3_, which implies possible agglomeration problems. Plot (C) shows the relations between fiber mesh size and nanofiller. Higher mesh sizes can cause higher void contents in the presence of MWCNT or Al_2_O_3_. The predicted versus actual plot in (D) demonstrates a good relationship thus supporting the model’s ability in the predicted void content behaviours.

#### Optimization variables

**Fig. 17:**
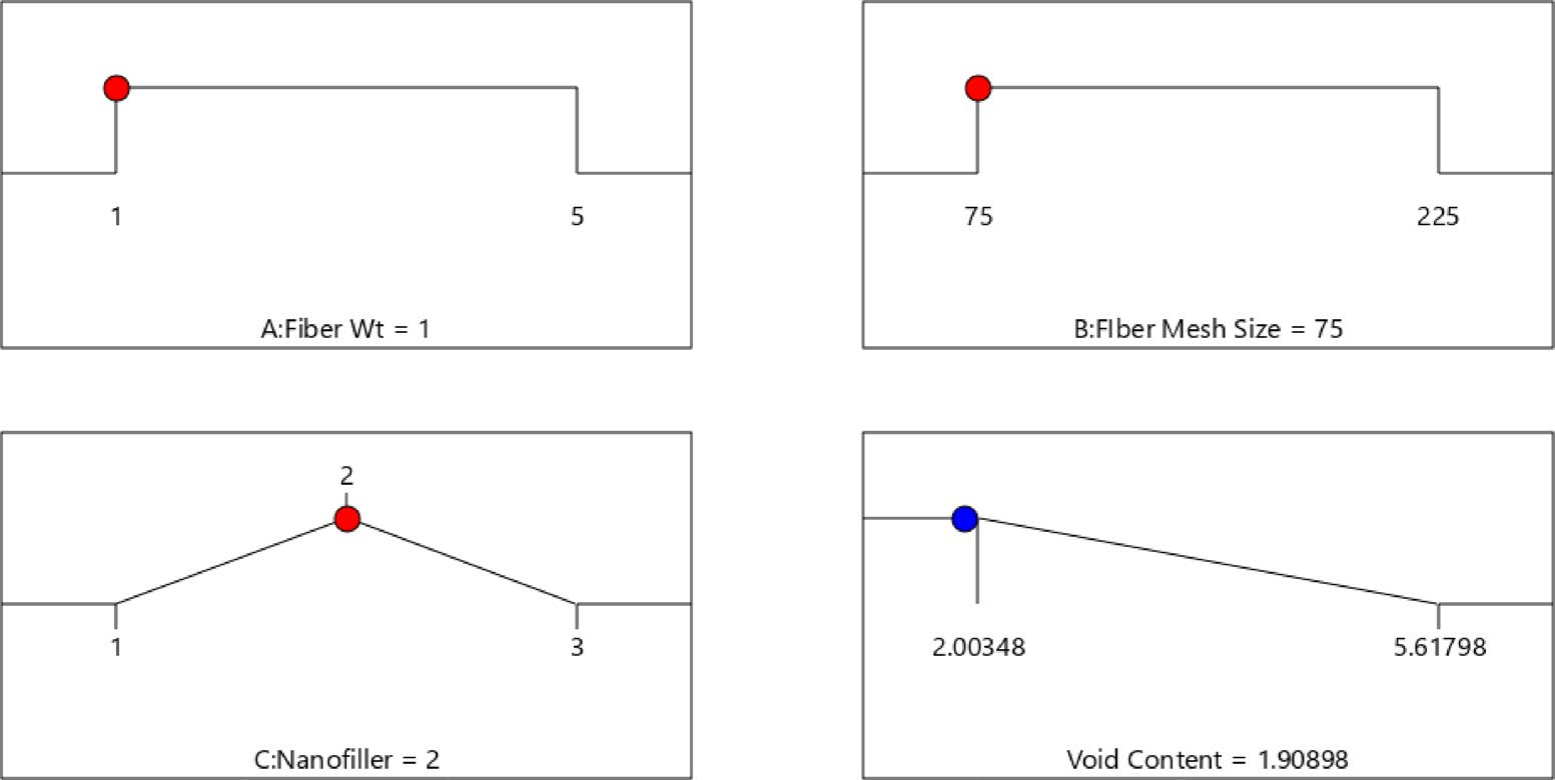
Optimized void content with input variables.

The main purpose of implementing response optimization techniques is to identify a more advantageous combination of experimental variables that can provide maximum response values. Following that, an extensive exploration into statistical analysis and optimization options is performed using the advanced features provided by Design Expert 13 software. The optimization results indicate that the ideal combination of process parameters for minimizing void content in bio-nanocomposites consists of a fiber weight of 1%, a fiber mesh size of 75 microns and the selection of nanofiller type 2, which corresponds to hexagonal boron nitride (h-BN) as shown in Fig. 17. This optimal condition yields a void content of 1.9%, which is close to the lower experimental limit of 2.0%, demonstrating model’s precision and reliability. The desirability function range of 0.99 confirms that the selected parameter combination achieves the best possible outcome within the defined constraints. The influence of fiber weight and mesh size suggests that lower values contribute to reduced void formation, while the choice of h-BN as the nanofiller highlights its effectiveness in minimizing voids compared to other fillers such as MWCNT and Al_2_O_3_. The comparison of predicted void content values with experimental data shows in Table 7 that the ANN model, with a smaller error of 1.15%, predicts more accurately than the RSM model, which exhibits a higher error of 4.69% under identical input conditions. ANN performs better than RSM in terms of prediction accuracy (1.15% vs. 4.69%) and R^2^ value (95.83 vs. 92.17), according to the comparative analysis shown in Table 8. But in contrast to the easier to understand and more straightforward RSM, ANN needs greater computing power and bigger datasets.

**Table 7:** Validation of void content.

S. no	BFF wt%	BFF mesh size (µm)	Nanofiller type (1 wt%)	Void content %	Error %
1	1	75	h-BN	2.003 (Experimental)	**–**
2	1	75	h-BN	1.909 (Optimized by RSM)	**4.92**
3	1	75	h.BN	1.98 (Optimized by ANN)	**1.14**

Significant values are in [bold].

**Table 8:** Comparative analysis of RSM and ANN.

Criteria	RSM	ANN
Prediction accuracy (error %)	4.69%	1.15%
R^2^ (coefficient of determination)	92.17	95.83
Computational time	Lower	Higher
Model complexity	Simple (Polynomial-based)	Complex (neural network)
Ease of interpretation	Easy	Moderate to difficult
Data requirement	Low (Design-based)	High (needs larger datasets)
Generalization capability	Limited to design space	Better generalization potential

## Conclusion

This study analyses the impact of nanofillers on the density and void content of epoxy/BFF composites. The findings indicate that owing to higher fiber content, both theoretical and measured densities are raised, together with the void content that therefore lowers the fiber volume fraction. Variations in density arise due to the difference in the densities of the nanofillers, i.e., Al_2_O_3_ has a higher density than h-BN and MWCNTs. The difference between the theoretical and experimental density points out the presence of voids which in turn negatively affects mechanical properties. This high fiber loading will promote agglomeration, which in turn will trap air and give rise to the increased void content, thus weakening interfacial bonding and letting cracks propagate. The ANOVA analysis verified that fiber content, mesh size and nanofiller type have a significant effect on void content. The quadratic regression model unravelled intricate interactions among parameters with much sensitivity for variation of void content. The use of NaOH-treated fibers enhances bonding at the interface by minimizing pull-out of fibers and debonding, while the micro-cracks from thermal mismatch continued to be an obstacle. The optimized ANN models validated with k-fold cross-validation indicate high predictability, low MAE and acceptable R^2^ values. Also, the response surface methodology (RSM) gives insight into optimizing processing parameters to reduce void content. Although this study successfully models void content, it needs to further investigate the direct relationship with mechanical and thermal performance. Also, processing conditions and long-term durability have to be viewed for the real-world applicability. In the future, physics-based simulations need to be combined with machine learning for improved void prediction and composite optimization. Hybrid approaches that combine FEA with ANN modelling may increase predictive accuracy. Expanding the dataset to include a wider range of fabrication conditions would further validate the model and enhance its reliability for aerospace, automotive and construction applications.

## Data Availability

The datasets used and/or analysed during the current study available from the corresponding author on reasonable request.
